# Clinical outcome after surgical management of spontaneous spinal epidural hematoma

**DOI:** 10.1007/s00701-024-06169-w

**Published:** 2024-06-28

**Authors:** Cédric Kissling, Levin Häni, Ralph T. Schär, Johannes Goldberg, Andreas Raabe, Christopher Marvin Jesse

**Affiliations:** Present Address: Department of Neurosurgery, Inselspital, Bern University Hospital, University of Bern, Bern, Switzerland

**Keywords:** Spinal epidural hematoma, Neurosurgical procedures, Laminotomy, Laminectomy

## Abstract

**Purpose:**

Spontaneous spinal epidural hematoma (SSEH) is a rare pathology characterized by a hemorrhage in the spinal epidural space without prior surgical or interventional procedure. Recent literature reported contradictory findings regarding the clinical, radiological and surgical factors determining the outcome, hence the objective of this retrospective analysis was to re-assess these outcome-determining factors.

**Methods:**

Patients surgically treated for SSEH at our institution from 2010 – 2022 were screened and retrospectively assessed regarding management including the time-to-treatment, the pre-and post-treatment clinical status, the radiological findings as well as other patient-specific parameters. The outcome was assessed using the modified McCormick Scale. Statistical analyses included binary logistic regression and Fisher’s exact test.

**Results:**

In total, 26 patients (17 men [65%], 9 women [35%], median age 70 years [interquartile range 26.5]) were included for analysis. The SSEHs were located cervically in 31%, cervicothoracically in 42% and thoracically in 27%. Twenty-four patients (92%) improved after surgery. Fifteen patients (58%) had a postoperative modified McCormick Scale grade of I (no residual symptoms) and 8 patients (31%) had a grade of II (mild symptoms). Only 3 (12%) patients remained with a modified McCormick Scale grade of IV or V (severe motor deficits / paraplegic). Neither time-to-treatment, craniocaudal hematoma expansion, axial hematoma occupation of the spinal canal, anticoagulation or antiplatelet drugs, nor the preoperative clinical status were significantly associated with the patients’ outcomes.

**Conclusion:**

Early surgical evacuation of SSEH generally leads to favorable clinical outcomes. Surgical hematoma evacuation should be indicated in all patients with symptomatic SSEH.

**Supplementary Information:**

The online version contains supplementary material available at 10.1007/s00701-024-06169-w.

## Introduction

Spontaneous spinal epidural hematoma (SSEH) is a rare entity of bleeding in the spinal epidural space without preceding surgical or interventional procedure. Its incidence is estimated to be 1 in 1′000′000 per year [11]. The exact etiology remains unclear; however, a rupture of the posterior epidural venous plexus seems to be the most likely cause of bleeding [3]. Further etiological factors might include minor trauma (especially in the presence of anticoagulation), hematological disorders causing a bleeding diathesis as well as spinal arteriovenous malformations and dural arteriovenous fistulas [9, 10]. SSEH usually manifests as severe back pain with an accompanying rapid deterioration of neurological function suggesting MRI diagnostics [3]. Higher patient age and the use of anticoagulants have been described as risk factors [1, 10, 15]. Typical management of SSEH consists of a (hemi-)laminectomy, laminotomy, or laminoplasty and evacuation of the hematoma [29].

Current evidence on the functional outcomes of SSEH and its determinants is scarce and mainly restricted to retrospective case series with a limited number of patients [1–10, 12–17, 19–23, 25–32].

The objective of this retrospective analysis was to assess the patient- and disease-specific determinants of functional outcome in a homogenous cohort of surgically treated SSEH patients.

## Material and methods

### Study design, inclusion and exclusion criteria

We conducted a monocentric, retrospective cohort study. We included patients diagnosed with SSEH and surgically treated at our neurosurgical unit from 2010 until 2022. Missing relevant data on the clinical status or the surgical procedure, missing radiological data and a refusal to provide general consent led to exclusion of patients. The local ethics committee approved the study (KEK Bern 2023–00770).

### Primary and secondary endpoints

The primary endpoint was the neurological outcome at last follow-up assessed as modified McCormick Scale grades. A “favorable” outcome was determined to be a modified McCormick Scale grade I (“neurologically intact, may have minimal dysesthesia”) for statistical analysis. Meanwhile, modified McCormick Scale grades II to V were considered an “unfavorable” outcome. An overview of the modified McCormick Scale is shown in Table 1 [18]. We assessed the association of neurological outcome and time-to-treatment after symptom onset categorized as < or > 12 h and < or > 24 h.

**Table 1:** The modified McCormick scale [18]

Grade	Modified McCormick scale
I	Intact neurologically, normal ambulation, minimal dysesthesia
II	Mild motor or sensory deficit, functional independence
III	Moderate deficit, limitation of function, independent with external aid
IV	Severe motor or sensory deficit, limited function, dependent
V	Paraplegia or quadriplegia, even with flickering movement

We secondarily assessed the pre-treatment clinical status including motor function scaled as Medical Research Council (MRC) grade 0 to 5, radiological features of SSEH and complications including re-operations in relation to neurological outcome. (For analysis, the weakest recorded motor function of the characteristic muscles in the neurological examination was used.)

### Surgical indication and follow-up

Emergency surgical hematoma evacuation via microsurgical (hemi-)laminectomies or laminotomies was indicated in patients presenting with an SSEH on MRI and sensory and/or motor deficits and/or spinal cord compression. A clinical follow-up was typically scheduled six weeks postoperatively. Patients with an SSEH on MRI without neurological deficits and without spinal cord compression were treated conservatively and were not included in this analysis.

### Radiological assessment

**Fig. 1:**
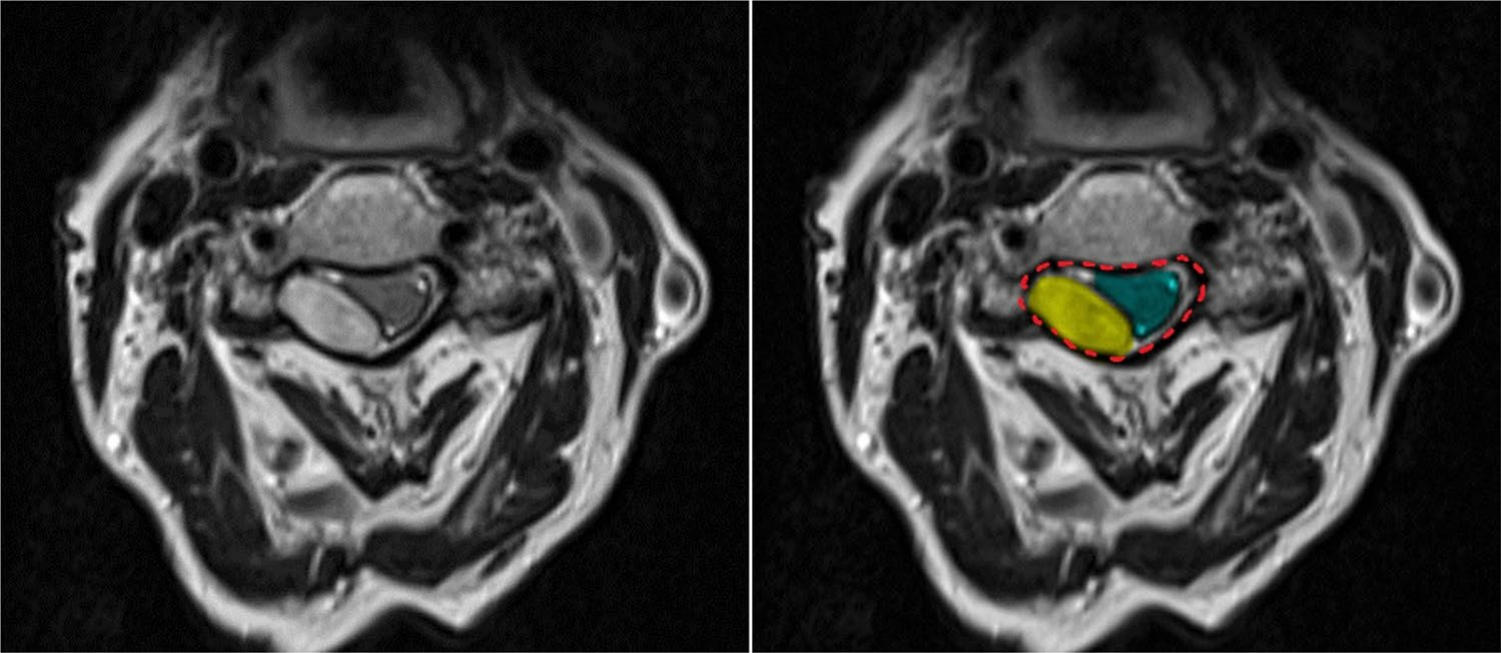
Axial T2-weighted MR image of one of the included patients demonstrating the radiological evaluation. The two images show the same axial slice. In the right image the spinal canal is outlined in a red dashed line, the spinal cord is highlighted in green, the SSEH is highlighted in yellow. The highlighted axial areas’ surfaces in mm^2^ were determined with the SECTRA IDS7 software

For radiological evaluation, Sectra Workstation IDS7 software (Version 24.2, Sectra AB, Linköping, Sweden) was used. The last spinal MRI before surgery was assessed for localization of the SSEH, the craniocaudal hematoma extension, the maximum axial area of the SSEH in the spinal canal in mm^2^, the maximum ratio of axial area of the spinal canal occupied by the SSEH in percent as well as preoperative intramedullary T2-weighted hyperintensities suggesting myelopathy. The SSEH extension was considered “short” if it spanned over ≤ 5 spinal segments and “long” if it spanned over > 5 spinal segments. The occupation of the spinal canal by the SSEH was considered “large” for > 50% occupation and “small” for < 50% occupation. Figure 1 shows an axial T2 MR image of one of the included patients demonstrating the appearance of the SSEH as well as the method of determining the axial area in mm^2^.

### Statistical analysis

All analyses were performed using IBM SPSS Statistics Version 28.0.1.1 (released 2021, Armonk, NY: IBM Corp.). Normal distribution of the continuous data was validated with a Shapiro–Wilk test. Univariate binary logistic regression was performed with the dichotomized McCormick Scale grades (I vs. II–V) as outcome variable. For categorical variables, a Fisher’s exact test was performed. A *p*-value < 0.05 was considered significant.

## Results

### Patient demographics

Twenty-six patients aged 70 years in median (range 19–85) diagnosed with an SSEH were included, 17 (65%) men and 9 (35%) women. The median BMI was 25.5 (range 17–41) kg/m^2^. Four patients were severely obese. The INR at admission was elevated (> 1.2) in 6 patients (23%). Eight patients (31%) were taking antiplatelet drugs, 6 patients (23%) were taking anticoagulants, no patient was taking both (Table 2, Appendix A).

**Table 2:** Patient demographics

	*N* (%)	Median	Interquartile range
Total No. of patients	26 (100)		
Sex			
Male	17 (65.4)		
Female	9 (34.6)		
Age	26 (100)	70	26.5
BMI	26 (100)	25.5	6.0
ASA	26 (100)	3	1
1 (I)	0 (0)		
2 (II)	7 (26.9)		
3 (III)	16 (61.5)		
4 (IV)	3 (11.5)		
5 (V)	0 (0)		
INR	26 (100)	1.005	0.148
Thrombocytes in G/L	20 (76.9)	205.5	89.5
Anticoagulants			
yes	6 (23.1)		
no	20 (76.9)		
Antiplatelets			
yes	8 (30.8)		
no	18 (69.2)		
Localization of spinal epidural hematoma			
Cervical	8 (30.8)		
Cervicothoracic	11 (42.3)		
Thoracic	7 (26.9)		
Number of segments involved	26 (100)	5.00	5.50
≤ 5 segments	16 (61.5)		
> 5 segments	10 (38.5)		
Maximal area of the hematoma in mm^2^	26 (100)	144.10	46.85
Highest axial ratio of spinal canal occupation by hematoma in %	26 (100)	56.27	14.26
≤ 50%	7 (26.9)		
> 50%	19 (73.1)		
Radiological signs of myelopathy	26 (100)		
yes	16 (61.5)		
no	10 (38.5)		
Preoperative modified McCormick Scale Grade	26 (100)	4.00 (IV)	1.25
1 (I)	2 (7.7)		
2 (II)	1 (3.8)		
3 (III)	3 (11.5)		
4 (IV)	11 (42.3)		
5 (V)	9 (34.6)		
Time to surgery			
< 12 h	18 (69.2)		
> 12 h	8 (30.8)		
< 24 h	23 (88.5)		
> 24 h	3 (11.5)		
Surgical technique			
Laminotomies	2 (7.7)		
Hemilaminectomies	19 (73.1)		
Laminectomies	5 (19.2)		
Duration of hospitalization in primary care in days	26 (100)	6.00	4.25
Last follow up in months	25 (96.2)	4.50	15
Complications			
Yes	6 (23.1)		
No	20 (76.9)		
Exit			
Home	8 (30.8)		
Other hospital	5 (19.2)		
Rehab	13 (50)		

### SSEH characteristics

**Fig. 2:**
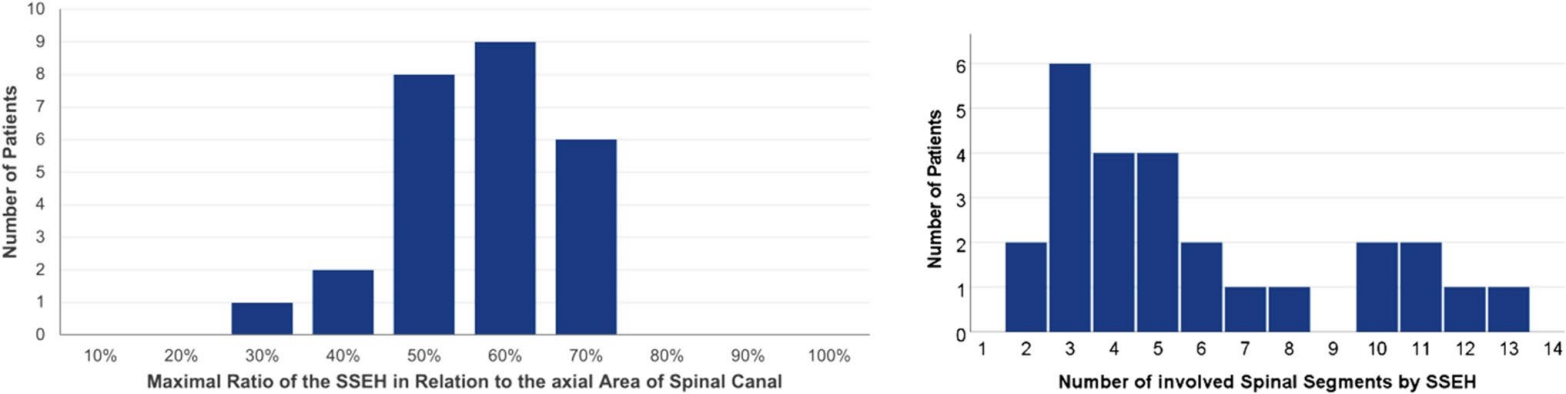
Radiological characteristics of the included SSEHs
(IV) Signs of myelopathy: Almost two thirds of the patients (*N* = 16; 62%) had intramedullary T2-weighted hyperintensities suggesting myelopathy on initial MRI.

SSEH was diagnosed by spinal MRI in all patients.

(I) Hematoma localization: In 8 patients (31%) the hematoma was located cervically, in 11 patients (42%) cervicothoracically and in 7 patients (27%) thoracically. None of the patients had a lumbar hematoma.
(II) Hematoma extent: The SSEH spanned across 5 spinal segments in median (range 2–13), while 16 patients (62%) had a “short” and 10 patients (38%) had a “long” SSEH.
(III) Hematoma occupation: When looking at the maximum axial occupation of the spinal canal by the hematoma, the values ranged from 31 to 74% with a median of 56% (Fig. 2).

### Clinical presentation and time-to-treatment

All patients initially presented with back pain. Twenty patients (77%) had a preoperative modified McCormick Scale grade of IV or V, 3 patients (12%) had a modified McCormick Scale grade of III and another 3 patients (12%) had a modified McCormick Scale grade of I or II. In 11 patients (42%) the worst motor score was MRC 0 (plegic), in 10 patients (39%) it was MRC 1–3 (severe paresis) and in 5 patients (19%) MRC 4 or 5 (slight or no paresis). Nineteen patients (73%) had sensory deficits. Eighteen patients (69%) underwent surgery within 12 h, 8 patients (31%) after more than 12 h. Of the latter, only 3 patients (12%) underwent surgery after more than 24 h. While most patients underwent hemilaminectomy (73%) for evacuation of the hematoma, laminotomy and laminectomy were more seldomly performed (8% resp. 19%). No patient developed a postoperative instability during the follow-up.

### Clinical outcome, discharge and complications

**Fig. 3:**
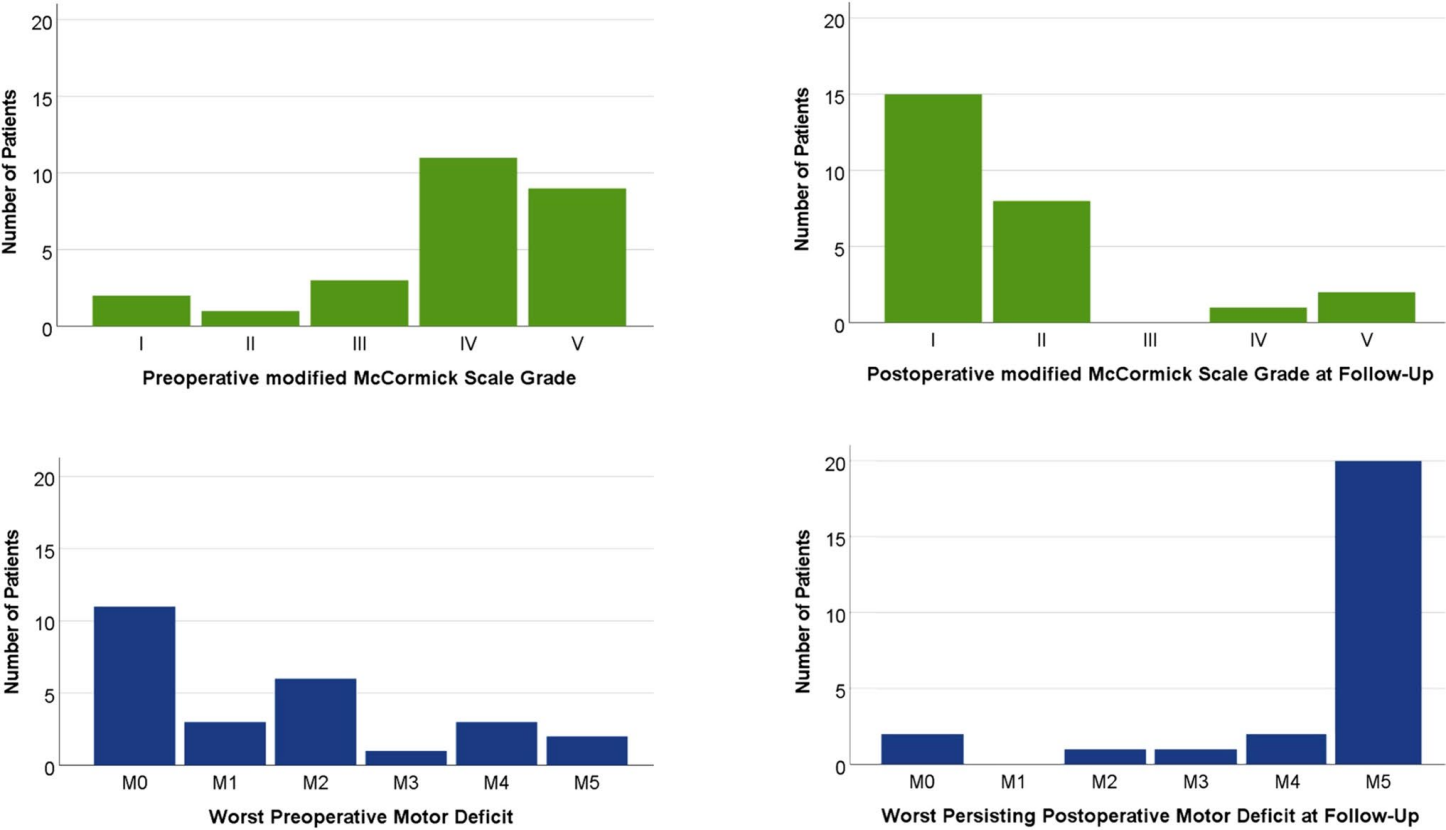
Evolution of modified McCormick Scale Grades (top) and worst MRC Grades (bottom) pre- (left) and postoperatively (right)

**Fig. 4:**
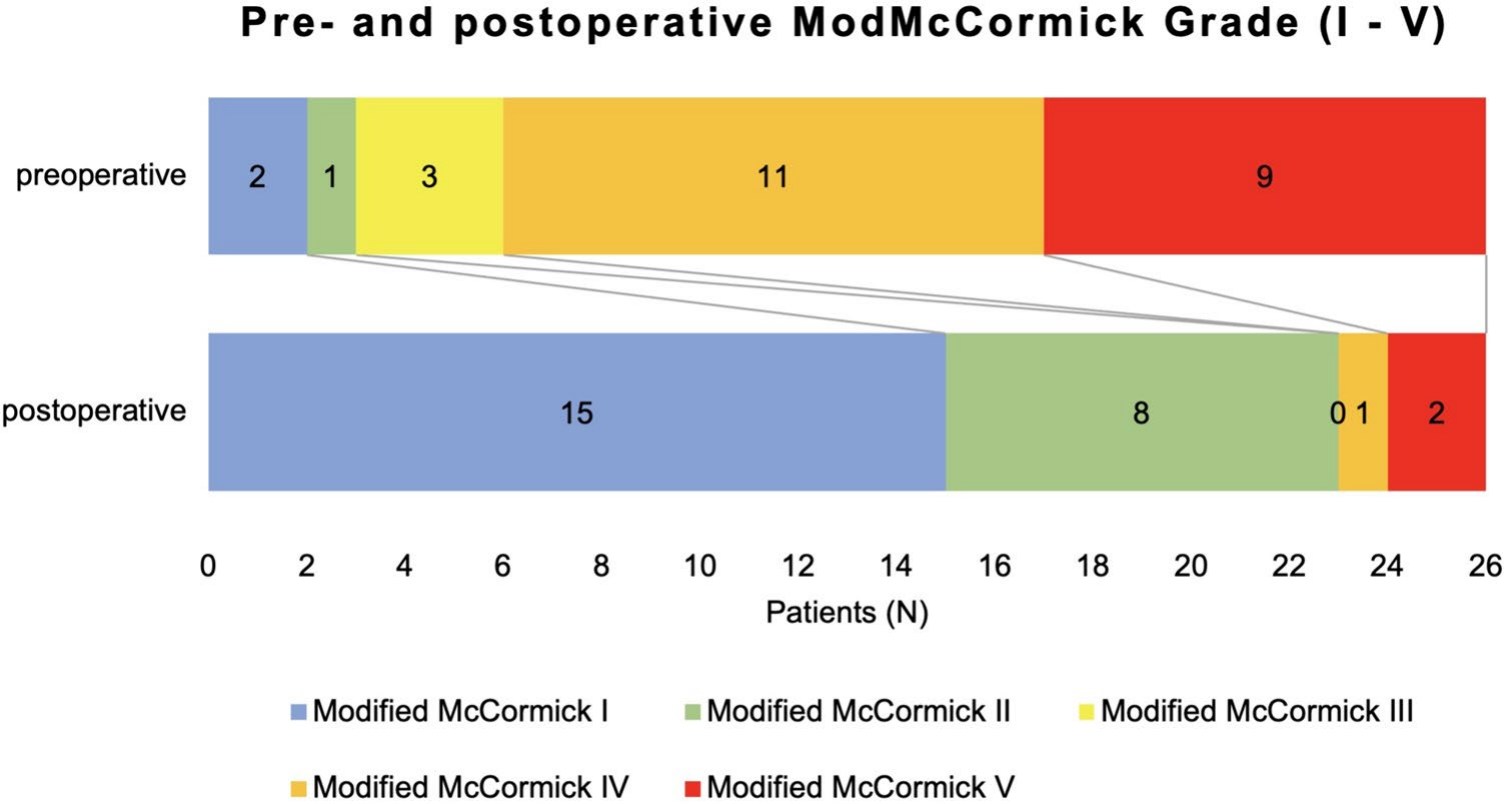
Shift in pre- and postoperative modified McCormick Scale Grades (“Grade I” translates into “neurologically intact, may have minimal dysesthesia”, “Grade II” into “mild motor or sensory deficit”, “Grade III” into “moderate motor or sensory deficit, limitation of function”, “Grade IV” into “severe motor or sensory deficit, loss of independence”, “Grade V” translates into “paraplegic” [18])

Postoperatively, 15 patients (58%) had a modified McCormick Scale grade of I, 8 patients (31%) of II, and 3 patients (12%) of IV or V (Fig. 3). Twenty-four out of 26 patients (92%) thus improved by at least one modified McCormick Scale grade (Fig. 4).

Six of 8 (75%) patients surgically treated *after* 12 h after symptom onset had a postoperative modified McCormick Scale grade of I, 2 of 8 (25%) patients of II. Nine of 18 (50%) patients surgically treated *before* 12 h after symptom had a postoperative modified McCormick Scale grade of I, 6 of 18 (33%) of II and 3 of 18 (17%) of IV or V. Eight patients (31%) were discharged home, 13 patients (50%) were transferred to a rehabilitation clinic and 5 patients (19%) were transferred to another hospital. Six patients (23%) developed a symptomatic rebleeding requiring a reoperation within few days after the first surgery. Neither vascular malformations in MRI or angiography nor an increased presence of anticoagulation or antiplatelet medication or underlying diseases with bleeding diathesis could be assessed in these patients. The median follow-up duration was 4.5 months (interquartile range 15 months).

The 3 patients with a modified McCormick Scale grade of IV or V at follow-up were all males. They underwent surgery after < 12 h. The canal occupation ratio of the cervicothoracic and thoracic SSEHs was in line with the average over the included 26 patients. Only one was on oral anticoagulation medication. The age distribution varied between 32 and 77, all 3 were severely obese with a BMI > 35.

### Outcome-determining factors

None of the clinical or radiological variables were significantly associated with the dichotomized modified McCormick Scale grade (II-V vs. I) at follow-up (Table 3, Appendix B).

**Table 3:** Binary logistic regression

Dependent variable	Postoperative modified McCormick Scale grades at follow-up dichotomized into II–V vs. I		
Independent variables ^*^	Odds ratio	95% CI of OR	Sig
1. max. craniocaudal expansion segments in** #**	1.087	0.856–1.381	0.492
2. max. ratio of SSEH in the spinal canal in %	1.022	0.946–1.105	0.576
3. anticoagulation (yes/no)	1.500	0.240–9.382	0.665
4. antiplatelet (yes/no)	0.750	0.136–4.133	0.741
5. preoperative MRI signs of myelopathy (yes/no)	0.600	0.121–2.973	0.532
6. symptom onset to surgery delay < 12 h	0.333	0.053–2.115	0.244
7. symptom onset to surgery delay < 24 h	0.650	0.051–8.226	0.739
8. sex ^†^	2.292	0.441–11.917	0.324
9. age	1.015	0.976–1.056	0.451
10. BMI	1.060	0.911–1.234	0.451
11. ASA category 1–2 (vs. 3–4)	2.250	0.346–14.611	0.396

*ASA* American society of anesthesiologists, *BMI* body mass index, *MRC* medical research council

^*^Analyses performed individually with the independent variables

^†^Female sex as reference category

## Discussion

Our analysis demonstrates a favorable overall clinical outcome of patients surgically treated for an SSEH. We did not find any significant association between the outcome and the analyzed clinical and radiological determinants.

Due to scarce data on SSEH, there are no management guidelines available [11]. Several publications however support an immediate evacuation of an SSEH in symptomatic cases [5, 8, 22, 29, 30]. Time-to-treatment, preoperative clinical status, use of anticoagulants, radiologically assessed factors such as the canal occupation ratio as well as the localization and expansion of the hematoma and evidence of myelopathy in the spinal cord on MRI imaging were correlated with clinical outcome [1, 3, 4, 8, 12, 22, 25, 29].

The satisfactory outcome with postoperative modified McCormick Scale grades of I or II in 88% and a functional improvement in 92% of patients in our cohort exceeds the results of a recent meta-analysis by Vastani et al. with 66% favorable outcomes [29]. Crucially, more than a third of the almost 300 patients included in their analysis underwent surgery after more than 24 h after symptom onset as opposed to 12% in our cohort, which is a possible explanation for this difference.

Our analysis showed no association of anticoagulation or antiplatelet medication with functional outcome. It however confirms their role as risk factor in the occurrence of SSEH as 53% of the patients in our study were receiving these medications [10]. Male sex was predominant with 65% in our cohort, while other similar analyses showed a more balanced sex distribution [2, 8].

The importance of time-to-treatment regarding the outcome of SSEH patients is contradictorily reported in two recent meta-analyses [3, 29]. While direct neural compression damage is not time-dependent, the potential secondary damage due to venous congestion, ischemia and edema might well be [22]. Along with this, many publications discussed a worse long-term outcome in patients with a longer time-to-treatment interval [8, 22, 25, 29, 30]. No such correlation could be confirmed in our cohort. In line with our results, Baeesa et al. recently reported favorable outcomes of 14 retrospectively analyzed SSEH patients despite a median time-to-treatment of 21 h [2]. While these results might suggest that the neurological outcome is not significantly worse after a longer time-to-treatment in SSEH patients, in our cohort only three of 26 patients (12%) underwent surgery later than 24 h after symptom onset. All three almost fully recovered with postoperative modified McCormick Scale grades of I and II. We however strongly believe that the neurological outcome is worse in patients with an SSEH after a certain period of ongoing symptoms. The shorter average delay to treatment might explain the higher proportion of favorable outcomes in our cohort compared to a meta-analysis [29].

The preoperative clinical status of SSEH patients was found to be crucial regarding the outcome in several publications [3, 13, 17, 22, 24, 29, 30, 32]. Interestingly, our analyses showed no significant correlation in this matter. Representatively, of 11 patients with a preoperative worst motor deficiency grade of MRC 0, six patients recovered to MRC 5 and two patients recovered to MRC 3. This suggests that even patients with pronounced neurological deficits have the potential for good recovery.

We further could not find a statistical correlation between radiological findings ((I) hematoma localization, (II) hematoma extent, (III) hematoma occupation of the spinal canal and (IV) signs of myelopathy) and clinical outcome. Especially, in contrary to a recent publication by Honda et al., the canal occupation ratio was not shown to be significantly correlated with the clinical outcome [12]. Moreover, of the 16 patients with radiological signs of myelopathy, ten patients had a favorable outcome. This might relativize the significance of these radiological signs regarding the clinical prognosis in SSEH.

In our opinion, the primary aim of surgical treatment of SSEH is to achieve decompression of the spinal cord, while the extent of bony resection is of secondary importance. Since most cases of SSEH are localized in the thoracic spine, postoperative instabilities are uncommon. In cases with extended SSEH in the cervical spine, laminoplasties may also be a valuable option.

The occurrence of rebleeding requiring re-surgery in six of the included 26 patients might support the theory of a fragile posterior epidural venous plexus as the primary etiological factor in SSEH [29]. Similarly, Yu et al. reported re-bleedings in 19 of 55 retrospectively analyzed SSEH patients [31]. They however did not further analyze these patients for etiological factors [31]. Importantly, of our six patients with a rebleeding, only one had an unfavorable outcome with a modified McCormick Scale grade of II. The initial SSEH was more extensive in these patients compared to the rest (ten vs. 4.5 segments in median). Other parameters such as INR and medication were not noticeably differing. Histological samples were not acquired.

Lastly, a subanalysis of the three patients with a bad outcome did not yield noticeably differing clinical or radiological findings compared to the rest of the cohort.

## Limitations

The main limitations of this study are the small cohort size and the retrospective nature of the analysis. Only three patients were surgically treated after more than 24 h after symptom onset. This could have led to a distorted assessment of the role of time-to-treatment regarding the outcome. It was therefore also not possible to assess patients who might suffer from symptoms due to a SSEH for too long and no longer benefit from surgery. While the surgical technique (laminotomy vs. (hemi)laminectomy) could not be shown to make a significant difference in clinical outcome, important details regarding the extent and the approach of the surgical treatment need yet to be addressed. With decompression of the neural structures through complete hematoma evacuation as the only surgical goal, we believe that the surgical invasiveness could be kept minimal. Especially, endoscopic approaches could be evaluated for surgical treatment of SSEH. Further, no conservatively treated SSEH patients were included in this analysis. It was thus not possible to draw any conclusions regarding patients who might not have required any surgical treatment at all. Moreover, the dates of the last follow-up varied between one week and over ten years postoperatively in our cohort which could have led to an underestimation of the clinical outcome in some patients due to incomplete recovery. Lastly, neither patient age nor BMI were corrected for in the outcome analyses.

## Conclusion

Early surgical evacuation of SSEH leads to favorable clinical outcomes. We found no association of time-to-treatment, severity of preoperative symptoms and radiological characteristics with the outcome. We thus propose surgical hematoma evacuation in all patients with neurologically symptomatic SSEH.

## Supplementary Information

Below is the link to the electronic supplementary material.

Supplementary file1 (DOCX 375 KB)

## Data Availability

Original uncoded research data is available to all researchers at any time. It will be safely stored at our institution for at least 10 years. Coded research data is available to everyone at any time. It will be safely stored at our institution for at least 10 years.

## Abbreviations

ASA I–IV: American society of anesthesiologists grade I–IV
BMI: Body mass index
INR: International normalized ratio
MRC 0-5: Medical research council grades 0–5
MRI: Magnetic resonance imaging
SSEH: Spontaneous spinal epidural hematoma
