# Supplementary material for: Clinical outcome after surgical management of spontaneous spinal epidural hematoma
Source: Acta Neurochir (Wien). 2024 Jun 28;166(1):277. doi: 10.1007/s00701-024-06169-w (PMC11211104; doi:10.1007/s00701-024-06169-w)
Supplement: Supplementary file 1 — Supplementary file1 (DOCX 375 KB) [file 701_2024_6169_MOESM1_ESM.docx]

**Supplementary Information**

**for**

**Clinical Outcome After Surgical Management of Spontaneous Spinal Epidural Hematoma**

Cédric Kissling, MD^1^, Levin Häni, MD^1^, Ralph T. Schär, MD^1^, Johannes Goldberg, MD^1^, Andreas Raabe, MD^1^, Christopher Marvin Jesse, MD^1^

^1^ Department of Neurosurgery, Inselspital, Bern University Hospital, University of Bern, Bern, Switzerland

**Corresponding Author:**

Cédric Kissling, MD

Universitätsklinik für Neurochirurgie

Inselspital

Rosenbühlgasse 25

CH-3010 Bern

Switzerland

[cedric.kissling@bluewin.ch](mailto:cedric.kissling@bluewin.ch)

**Appendix A:** Graphical representation of patient age and BMI distribution as well as clinical, radiological and procedural details


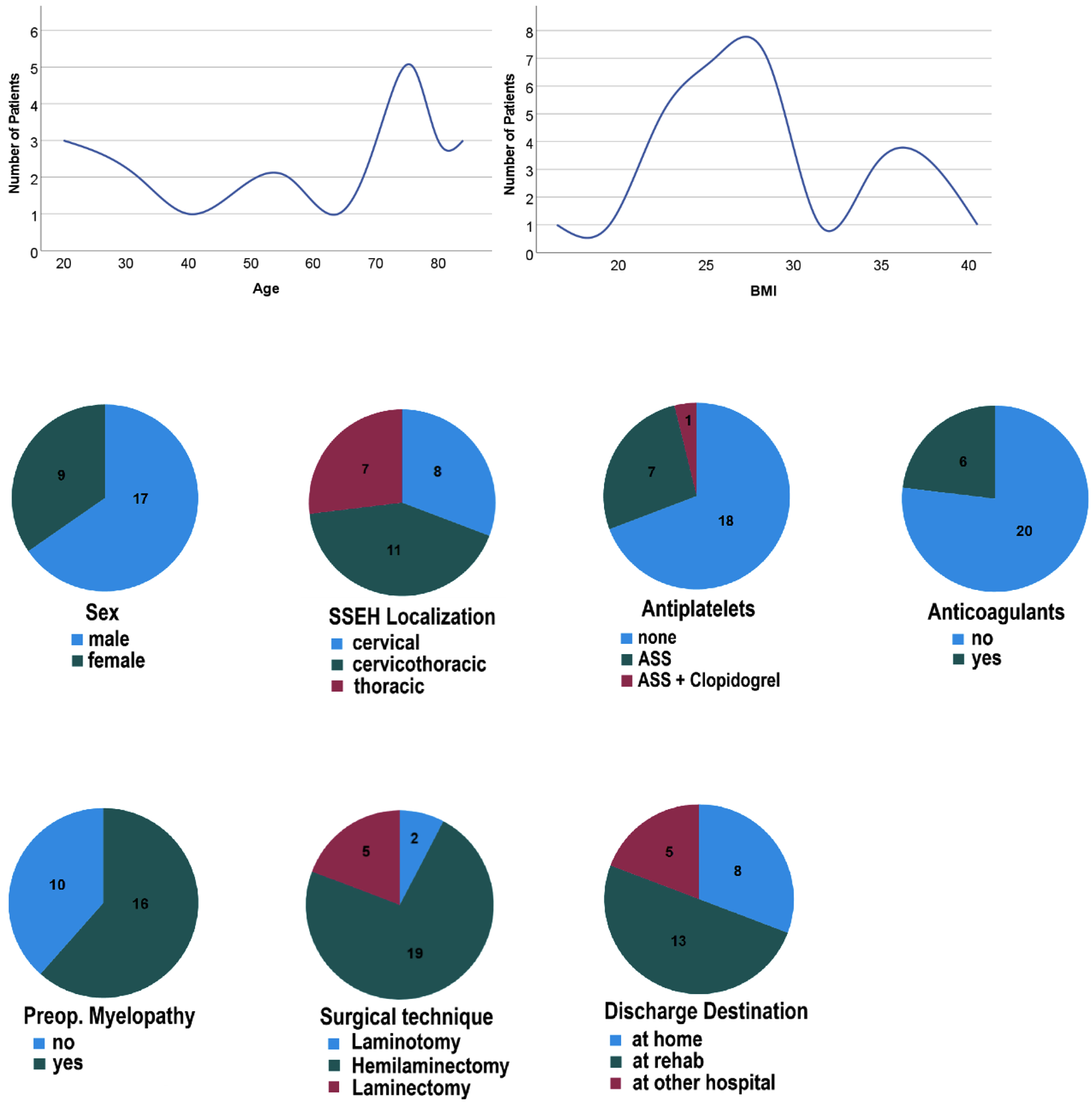


**Appendix B**

| **Table**: Two-sided exact Significance in Fisher’s Exact Test | |
| --- | --- |
| **Variable in columns** | **Postoperative modified McCormick Scale grades at  Follow-Up dichotomized into II-V vs. I** |
| **Variables in rows:** | Two-sided exact Significance in Fisher’s Exact Test |
| 1. **max. craniocaudal expansion in # of segments dichotomized into ≤ 5 vs. > 5** | 1.00 |
| 1. **max. ratio of SSEH in the spinal canal dichotomized into > 50% vs. < 50%** | 0.658 |
| 1. **worst preoperative motor grade dichotomized into**  **≤ MRC 3 vs. ≥ MRC 4** | 0.053 |
| 1. **anticoagulation (yes/no)** | 1.00 |
| 1. **antiplatelet (yes/no)** | 1.00 |
| 1. **preoperative MRI signs of myelopathy (yes/no)** | 0.689 |
| 1. **symptom onset to surgery delay dichotomized into  > 12h vs. < 12h** | 0.395 |
| 1. **symptom onset to surgery delay dichotomized into  > 24h vs. < 24h** | 1.00 |
| 1. **ASA category dichotomized into 1-2 vs. 3-4** | 0.658 |
| *^*^ analyses performed individually with the independent variables*  ASA: American Society of Anesthesiologists; MRC: Medical Research Council | |
